# An *Aloe Vera*-Based Cosmeceutical Cream Delays and Mitigates Ionizing Radiation-Induced Dermatitis in Head and Neck Cancer Patients Undergoing Curative Radiotherapy: A Clinical Study

**DOI:** 10.3390/medicines4030044

**Published:** 2017-06-24

**Authors:** Suresh Rao, Sanath Kumar Hegde, Manjeshwar Poonam Baliga-Rao, Princy Louis Palatty, Thomas George, Manjeshwar Shrinath Baliga

**Affiliations:** 1 Department of Radiation Oncology, Mangalore Institute of Oncology, Mangalore 575002, India; raos_64@yahoo.com (S.R.); Sanathkumarhegdemio@yahoo.com (S.K.H.); 2 Department of Pharmacy Division, Mangalore Institute of Oncology, Mangalore 575002, India; poonam.baliga.rao@gmail.com; 3 Department of Pharmacology, Father Muller Medical College, Kankanady, Mangalore 575002; India; drprincylouispalatty@gmail.com (P.L.P.); jeffthomasgeorge@gmail.com (T.G.)

**Keywords:** radiation dermatitis, radiodermatitis, head and neck cancer, *Aloe vera*, Radiation Therapy Oncology Group (RTOG)

## Abstract

**Background:** This study was planned to evaluate the efficacy of topical application of an *Aloe vera*-based cream (AVC) for the prevention of ionizing radiation (X ray)-induced dermatitis in head and neck cancer patients requiring therapeutic radiation treatment (>62 Gy). **Methods:** From July 2012 to December 2012, a total of 60 head and neck cancer patients requiring curative radiotherapy (RT) of more than 66 Gy were prospectively enrolled and treated with AVC or a comparator Johnson’s Baby Oil (JBO). Acute skin reaction was monitored and classified according to the Radiation Therapy Oncology Group (RTOG) four-point rating scale on a weekly basis. **Results:** The results indicate that there was a statistically significant delay in the incidence (*p* = 0.04) of dermatitis at week three in the AVC application group. Application of AVC reduced the incidence of Grade 1, 2, and 3 dermatitis at subsequent time points, while Grade 4 dermatitis was not seen in either cohort. The other most important observation was that the continued application of AVC two weeks after the completion of RT was effective in reducing the average grade of dermatitis and was statistically significant (*p* < 0.02). **Conclusions**: Prophylactic use of an AVC-based cream is thus effective in delaying radiation dermatitis in head and neck cancer.

## 1. Introduction

Head and neck cancers (H & N) are globally the sixth most common cancer and a major health burden [1]. The most important aspects of H & N cancers are that they are a heterogeneous group and include tumors growing in the oral cavity, pharynx, and larynx regions [2,3]. From a therapeutic perspective, surgery, chemotherapy, and radiotherapy are the most important treatment strategies and are used either alone or in combination to obtain complete remission and cure [3]. Of these, radiotherapy is extremely useful in the treatment of inoperable tumors and is used either alone or in combination with a small dose of chemotherapy (termed chemo-irradiation), usually cisplatin or carboplatin [3,4,5]. However, curative radiotherapy causes radiation-induced-dermatitis (also known as radiodermatitis) in most patients [6,7,8,9,10,11]. Conventionally, steroidal, non-steroidal, and metallic topical preparations and dressings are used to mitigate radiodermatitis [12,13]. However, in the recent past, skin care products containing extracts of *Aloe vera* [14,15,16], silymarin [17], and marigold [18] have been reported to be beneficial. Of these, *Aloe vera* has been investigated in detail in patients undergoing curative radiotherapy for their breast cancer [14,15,16]. 

The plant *Aloe vera* is one of the most well-researched plants in skin care and studies have shown it to be useful in the treatment of cuts, abrasions, burns, sunburns, and eczema [19]. Experimental studies with laboratory animals have also shown it to be effective in reducing UV radiation-induced skin erythema, inflammation [20], contact hypersensitivity (CHS), and delayed-type hypersensitivity (DTH) [21]. *Aloe vera* prevents chemical-induced skin carcinogenesis [22,23] and is also reported to be effective in preventing senescence [24], healing psoriasis [25], and enhancing the healing of partial thickness burn wounds [26]. 

*Aloe vera* has been investigated for its protective effects against radiodermatitis in women undergoing curative treatment for breast cancer, and the observations are contradictory [15,16,19]. From a dermatological perspective, the region of the head and neck is more susceptible to a severe degree of dermatitis as the region has skin folds and an undulated surface. This leads to diminished aesthetic appearance, is associated with pain, and affects the quality of life of the patient. For an agent to be beneficial, it is advisable that its pharmacological effects be studied in various anatomical areas. Considering this, the present study was carried out in patients requiring curative radiotherapy for their H & N cancers. 

## 2. Materials and Methods

This was a single-center, investigator-blinded random sampling study and was conducted between July 2012 and December 2012 in the Department of Radiation Oncology at Mangalore Institute of Oncology, Pumpwell, Mangalore, India. The subjects comprised of histopathologically confirmed adult patients with head and neck cancer scheduled to receive radiotherapy or chemoradiotherapy. H & N cancer cases that had radical surgery prior to six weeks at the start of radiation treatment were also included. The other most important criteria were that only patients with a Karnofsky Performance Scale [27] of above 70 (patient can care for self but is unable to carry on normal activity or do active work) were included. 

The exclusion criteria included patients less than 18 years of age; women with positive pregnancy test or lactating; patients on high doses of non-steroidal anti-inflammatory drugs; those with poor oral hygiene and significant co-morbidity (such as poorly controlled diabetes mellitus, hypertension, schizophrenia, bipolar disorders, and severe depression). Patients who had had oral surgery within the past six weeks, chemotherapy within the last eight weeks, or who had previously been treated with radiotherapy for cancers in the H & N regions were also excluded. 

The study was approved by the Institutional Ethics Committee and was carried out in accordance with the guidelines of the Helsinki Declaration. Each patient and their caregivers had the treatment schedule, the benefits, and the possible adverse effects of the study explained by a trained professional in a private counseling room of the institute. They were also informed about their right to withdraw during the course of the study and that this would not affect their proposed treatment. Queries from both patients and caregivers were answered and written consent was obtained from all willing patients.

### 2.1. Patients and Methods

Willing patients fulfilling the inclusion criteria were randomized into either of two groups using opaque envelopes by an investigator unaware of the patient’s details. Patients allocated to Group 1 received JBO (Johnson’s Baby Oil), while Group 2 received AVC (*Aloe vera*-based cream).

### 2.2. Radiation Therapy Treatment

All patients who participated in this study received external irradiation from a linear accelerator (Varian, Model Unique Performance, Palo Alto, CA, USA) at a maximum energy level of 6 MV at a dose rate of 300 MU/min. All planned fields were treated every day with no more than one fraction of 2 Gy per day, five times a week, at the same time of day without any intended gaps, for a planned target dose of 66 Gy (seven consecutive weeks). Whenever chemo-irradiation was planned, carboplatin infusion (70 mg/m^2^/day intravenous) was administered on a weekly basis three hours before exposure to the first weekly radiation [28]. 

### 2.3. Application of JBO and AVC

AVC (Elovera, Glenmark, Mumbai, India) and JBO (Johnson & Johnson, Mumbai, India) were procured from designated dealers of the company and provided to the patient/ caregiver according to the randomization by one of the investigators. To avoid batch to batch variation a single lot of the required amount of the cream and oil were procured before the start of the study.

Prior to the start of radiation, the volunteers and their caregivers were also taught the correct way of applying the JBO (5 mL) or AVC (5 g) by one of the investigators. During the course of the treatment, the patients were also instructed to apply the cream five times a day (two hours before, immediately after, two hours after, four hours after, and six hours after radiotherapy). The application of the cream or oil was recommended to be performed with the ventral surface of the fingers using a rotary motion of the fingers with light pressure to the skin. The oil/cream was massaged into the skin until the surface of the skin no longer felt greasy. No other prophylactic creams, lotions, or gels were recommended/allowed during the study period. When moist desquamation occurred, the topical application of the JBO or AVC cream was discontinued in the affected area and 1% gentian violet paint was applied until the wound healed. However, the patients were advised to continue with the topical preparation of the JBO and the cream in the area of radiation that was free of moist desquamation. To preserve the single blinding, patients were instructed not to use the agent 2 h or less before an irradiation session or before evaluation by the doctor.

Patients of both cohorts were advised to use lukewarm water and a gentle detergent to wash and told not to shampoo their hair or scratch the irradiated skin; men were advised against using razor blades. They were instructed to use a head scarf or shawl to cover the skin from direct sunlight, pat the skin dry with a soft towel after washing, and keep the irradiated skin dry. During the course of the treatment, one of the investigators checked for the usage of the cream/oil on a weekly basis and repeatedly instructed volunteers in both groups to adhere to the application of the oil/cream. As one point of application was immediately after irradiation, it was easy for the investigators to keep a record of the patient’s adherence to oil/cream. A carefully planned diet was provided for all the patients.

### 2.4. Patient Evaluation

The patients of both groups were assessed for radiation-induced dermatitis by an experienced physician unaware of the experimental classification and dermatological treatment. The assessment was undertaken every week on Fridays in accordance with the criteria of the Radiation Therapy Oncology Group/European Organization for Research and Treatment Cancer (RTOG/EORTC) [29]. The criteria were as follows: Grade 0: no skin rending, ulceration, inflammation or damage; Grade 1: Faint erythema or dry desquamation; Grade 2: Moderate to brisk erythema, patchy moist desquamation mostly confined to skin folds and creases, moderate edema; Grade 3: radiation dermatitis consists of moist desquamation ≤1.5 cm diameter, other than skin folds or creases and bleeding induced by minor trauma or abrasion; and Grade 4: skin necrosis or ulceration of full thickness dermis; spontaneous bleeding from the involved site. This score makes a useful distinction between faint erythema and tender, bright erythema, as well as between patchy and confluent moist desquamation, and is probably the most widely used in practice and research [10,11]. At every investigation, the investigator considered the score for the worst toxicity in the treatment. 

### 2.5. Statistical Analysis 

The Student’s *t*-test was used to assess the difference in the degree of dermatitis at each time point, along with the delay in incidence and the number of dermatitis, while the *x*^2^ test was used to compare the total incidence of dermatitis using the online Vassar stat and Social Science Statistics program. A *p* value <0.05 was considered significant.

## 3. Results

**Figure 1:**
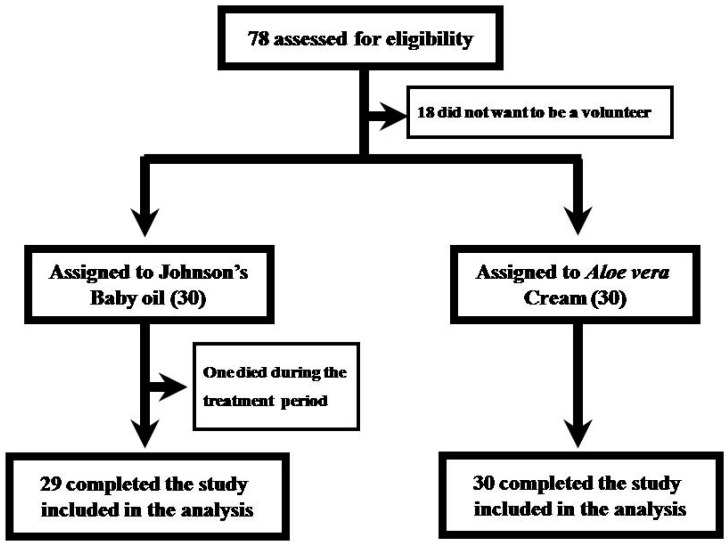
Patient flow in the randomized controlled study.

The demographic information and the cancer details are represented in Table 1, respectively. With respect to the age, the mean age for the JBO group was 55.2 ± 9.66 (range 30–73), while for AVC it was 55.9 ± 8.99 (range 35–71) and was statistically not significant. The male to female ratio was 24:6 in the JBO and 26:4 for the AVC group, respectively. Tumor characteristics in the two groups are represented in Table 1. Of the 60 patients enrolled, 59 were available throughout the study period until two weeks post-irradiation (Figure 1). One patient in the JBO group died of cancer during the second week of treatment, giving an effective number of 59 for the study and 29 for the JBO arm of the cohort. 

**Figure 2:**
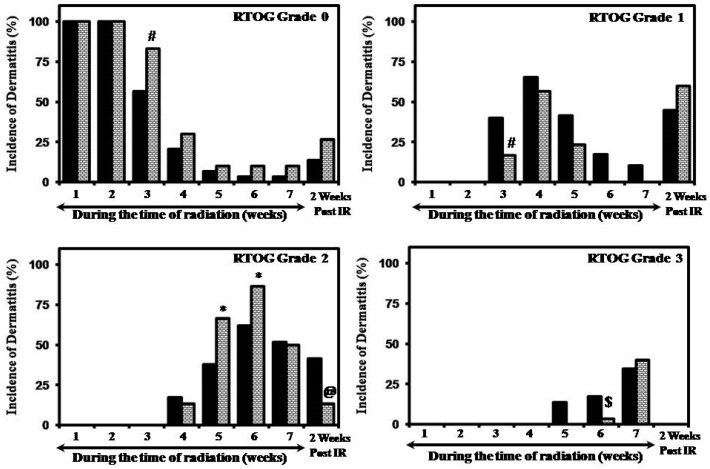
Incidence of dermatitis through the treatment period and two weeks after completion of treatment (*x*^2^ test @ = *p* < 0.02; * = *p* < 0.03; # = *p* < 0.04; $ = *p* = 0.07 (not significant)).

**Figure 3:**
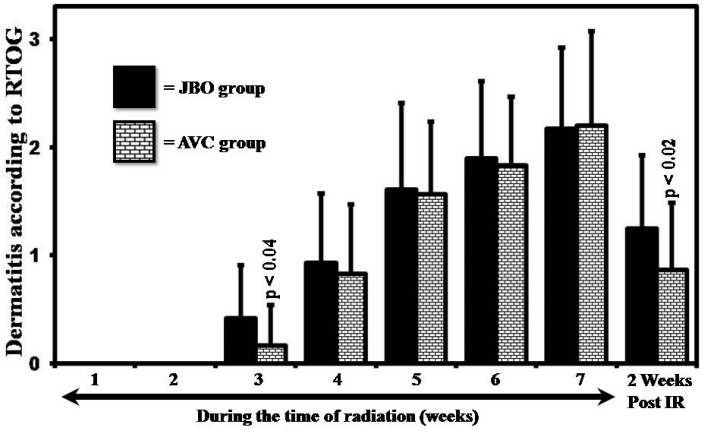
Differences in the degree of radiation-induced dermatitis in the two groups during and after completion of radiation. Solid black bars represent JBO, while bricked bars represent AVC with the standard deviation. The lines above the bars are the standard deviation.

The radiation dermatitis was evaluated in accordance with the RTOG guidelines and at the end of the first and second week; patients in both groups had normal skin and did not show any indications of RTOG-specified radiodermatitis (Figure 2 and Figure 3). The incidence of dermatitis after the second week was as follows in the JBO group: 41.4% (12/29 week 3), 82.8% (24/29 week 4), 93.1% (27/29 week 5), 96.6% (28/29 week 6), 96.6% (28/29 week 7) and 86.2% (25/29) at two weeks post treatment; while in the AVC it was 16.7% (5/30 week 3); 70% (21/30 week 4); 90% (27/30 week 5); 90% (27/30 week 6); 90% (27/30 week 7); and 73.3% (22/30 week 8) at two weeks post-treatment. Analysis also showed that at the end of the study 96.55% (28/29) and 90.00% (27/30) of volunteers in the JBO and AVC groups developed dermatitis.

The incidence of Grade 1 dermatitis was seen in the third week: 41.40% (12/29) of the volunteers in the JBO group and 16.66% (5/30) in the AVC group, respectively, developed dermatitis, which was statistically significant (*x*^2^; *p* = 0.04) (Figure 2). Grade 2 dermatitis was first seen at the end of week 4 in both groups: 17.24% (5/29) and 13.33% (4/30) in JBO and AVC, respectively (Figure 2). Continuation of the radiation treatment increased the incidence and degree of dermatitis (Grades 2 and 3) in both groups (Figure 2 and Figure 3).

The highest dermatitis observed in the study was Grade 3; 34.48% (10/29) and 40.00% (12/30) developed it in the JBO and AVC cohorts, respectively, at week seven (Figure 2). In the JBO group, Grade 3 was observed as follows: 13.79% (4/29) at week 5; 17.25% (5/29) at week 6; and a peak of 34.48% (10/29) at week 7 (Figure 2). Application of AVC reduced the cases of patients with Grade 3 radiodermatitis: 3.33% (1/30) and 40.00% (12/30) incidence were observed at weeks 6 and 7, respectively (Figure 2). In this study, none of the 59 evaluable individuals developed Grade 4 dermatitis.

Observation of the incidence and grade of dermatitis two weeks after the completion of the treatment showed that when compared to the JBO the average grade of dermatitis was lower in the AVC group (1.2 ± 0.67 vs. 0.87 ± 0.6) and was statistically significant (*p* < 0.02) (Figure 3). The cohort applying AVC had a lower incidence of Grade 2 dermatitis [41.38% (12/29) vs. 13.33% (4/30)], which was statistically significant (*p* < 0.02). 

## 4. Discussion

Radiodermatitis is an inevitable side effect observed during the course of ionizing radiation treatment; its mitigation is vital for the uninterrupted completion of the planned treatment [12,30]. From this study two important points can be inferred. The first is that applying an *Aloe vera*-based cream delays the incidence (Figure 2) and mitigates the grade (Figure 3) of dermatitis (when evaluated from the initiation of the treatment). The second observation is that continuing *Aloe vera* application after completion of the treatment enhances recovery by reducing the average degree of dermatitis (1.2 ± 0.67 vs. 0.87 ± 0.6) (Figure 3) and incidence of Grade 2 dermatitis (41.38% vs. 13.33%) (Figure 2). 

Previous studies have shown that *Aloe vera* gel, when combined with mild soap, mitigates dermatitis in women undergoing radiation treatment for breast cancer [31]. However, the observation that an *Aloe vera*-based cream delays the incidence of dermatitis (when evaluated from the initiation of the treatment) is novel and indicates the usefulness of *Aloe vera* in enhancing the healing of radiation dermatitis. Together, both these observations indicate the usefulness of *Aloe vera* in delaying and mitigating dermatitis and promoting recovery.

The mechanism by which radiodermatitis develops is multifaceted and includes myriad overlapping events [32,33,34,35,36,37]. At a molecular level, exposure to X-rays, a low LET ionizing radiation, causes cell damage and death by generating free radicals and inducing DNA strand breaks [32,33]. *Aloe vera* is reported to be a potent antioxidant and inhibits TPA-induced ear edema and tumor promotion in mouse skin [38]. Further experiments with laboratory rats have also shown that *Aloe saponaria Haw*, a sister species of *Aloe vera*, was effective in decreasing UVB-induced nociception (allodynia and hyperalgesia), leukocyte infiltration, inflammation, and edema [35]. Additionally, methanol extracts of *Aloe arborescens* were effective in scavenging reactive oxygen and protecting DNA [36]. 

*Aloe vera* has been investigated for its radioprotective properties; studies have also shown that the *Aloe vera* leaf extract protects mice against radiation-induced sickness and lethality [37]. In this study it was observed that at week 6, the cohorts where AVC was applied had a lower incidence of Grade 3 dermatitis [17.25% (5/29) vs. 3.33% (1/30) Figure 2]. Although not significant (x^2^; *p* = 0.07), the result indicates that AVC was effective in delaying the development of Grade 3 dermatitis. Acute radiodermatitis is a primarily inflammatory reaction and pro-inflammatory cytokines, which are rapidly activated after tissue irradiation, play a major role in the radiation dermatitis [9,39]. Previous studies have shown that *Aloe vera* possesses anti-inflammatory effects that can be mediated by modulating the levels of cytokines and the relevant signal transduction pathway in various study models [40,41]. 

The other important observation was that application of AVC after the completion of radiation treatment was effective in reducing the incidence of Grade 2 dermatitis [41.38% (12/29) vs. 13.33% (4/30); p < 0.02; Figure 2) and the mean dermatitis score (1.2 ± 0.67 vs. 0.87 ± 0.6; p < 0.02; Figure 3). Scientific studies have shown *Aloe vera* to be effective in enhancing burn wound healing and a similar mechanism may be occurring here. Both these aspects needs to be validated and studies are being planned to validate the hypothesis. *Aloe vera* has been used as a wound healing agent for centuries [42,43,44,45,46,47,48,49] and, to substantiate our observations, studies have also shown that *Aloe vera* gel was effective in mitigating the adverse effects [48] and enhancing the wound healing process [49] in laboratory animals exposed to ionizing radiation; it also was effective at healing burn wounds in laboratory rats [46] and in humans [26]. In lieu of all these observations, and considering our observations, it can be postulated that *Aloe vera* reduced radiation-induced dermatitis, possibly by triggering anti-inflammatory and antioxidant responses, modulating cytokines, and enhancing the wound healing process. Mechanistic studies are being planned to validate our hypothesis with suitable models of study. 

## Figures and Tables

**Table 1:** Patient and tumor characteristics.

Details	JBO (Johnson’s Baby Oil)	AVC (*Aloe Vera*-Based Cream)
*Age (Yrs)*	55.2 ± 9.66	55.9 ± 8.99
*Gender*		
Male	24	26
Female	6	4
*Tumor Site*		
Alveolus	2	1
Buccal mucosa	2	1
glottis	1	2
larynx	2	3
Maxilla	3	3
Pyriform sinus	1	2
Pharynx (Oro and Hypo)	3	1
Posterior cricoid	1	1
Retromolar trigone	1	1
Supraglottis	5	5
Tongue	6	7
Vocal cord	3	3
*TNM stage*		
*Primary*		
T_1_	1	3
T_2_	15	14
T_3_	12	10
T_4_	1	3
T_X_	1	-
*Regional nodes*		
N_0_	6	7
N_1_	12	16
N_2_	4	3
N_2a_	-	2
N_2b_	4	1
N_2c_	3	-
N_3_	2	-
N_X_	-	-
*Metastasis*		
M_0_	28	29
M_X_	2	1
*Radiation details*		
Dose of radiation (Gy)	69.0 ± 1.9	67.0 ± 3.7
Total Fraction (in 7 weeks)	34.3 ± 0.7	33.7 ± 1.9
